# Serum Cytokine Alterations Associated with Age of Patients with Nephropathia Epidemica

**DOI:** 10.1155/2022/4685288

**Published:** 2022-01-11

**Authors:** Venera Shakirova, Ilseyar Khaertynova, Maria Markelova, Rachael Tarlinton, Jerzy Behnke, Ekaterina Martynova, Ekaterina Garanina, Albert Rizvanov, Svetlana Khaiboullina

**Affiliations:** ^1^Department of Infectious Diseases, Kazan State Medical Academy, Kazan, Tatarstan 420012, Russia; ^2^Institute of Fundamental Medicine and Biology, Kazan Federal University, Kazan 420008, Russia; ^3^School of Veterinary Medicine and Science, University of Nottingham, Loughborough LE12 9RH, USA; ^4^School of Life Sciences, University of Nottingham, University Campus, Nottingham NG7 2RD, USA

## Abstract

Nephropathia epidemica (NE) is a zoonotic disease caused by hantaviruses transmitted from rodents, endemic in the Republic of Tatarstan, Russia. The disease presents clinically with mild, moderate, and severe forms, and time-dependent febrile, oliguric, and polyuric stages of the disease are also recognized. The patient's cytokine responses have been suggested to play a central role in disease pathogenesis; however, little is known about the different patterns of cytokine expression in NE in cohorts of different ages and sexes. Serum samples and clinical records were collected from 139 patients and 57 controls (healthy donors) and were used to analyze 48 analytes with the Bio-Plex multiplex magnetic bead-based antibody detection kits. Principal component analysis of 137 patient and 55 controls (for which there was full data) identified two components that individually accounted for >15% of the total variance in results and together for 38% of the total variance. PC1 represented a proinflammatory TH17/TH2 cell antiviral cytokine profile and PC2 a more antiviral cytokine profile with patients tending to display one or the other of these. Severity of disease and stage of illness did not show any correlation with PC1 profiles; however, significant differences were seen in patients with high PC1 profiles vs. lower for a number of individual clinical parameters: High PC1 patients showed a reduced number of febrile days, but higher maximum urine output, higher creatinine levels, and lower platelet levels. Overall, the results of this study point towards a stronger proinflammatory profile occurring in younger NE patients, this being associated with markers of acute kidney injury and low levels of high-density cholesterol. This is consistent with previous work indicating that the pathology of NE is immune driven, with an inflammatory immune response being associated with disease and that this immune response is more extreme in younger patients.

## 1. Introduction

Nephropathia epidemica (NE) is a mild form of hemorrhagic fever with renal syndrome (HFRS), a febrile zoonotic disease characterized by hemorrhages and renal pathology [1]. The disease has an acute onset with fever, headache, nausea, vomiting, hematuria, and back pain [2–4]. Laboratory findings typically include thrombocytopenia, leukocytosis, decreased CD4 : CD8 ratio, increased B lymphocyte counts, and increased serum creatinine levels [4–9]. Acute kidney injury is the major pathological finding and described in all cases. In severe cases, kidney failure can develop [10]. NE presents in three forms: mild, moderate, and severe [11, 12]. Each form of the disease progression includes febrile, oliguric, and polyuric periods, followed by convalescence. The severe form of NE is characterized by headache, vomiting, high fever (over 39.5°C), and acute kidney injury. The most prominent clinical features of this form of NE are hemorrhagic symptoms including petechial, nasal, and internal bleeding [11–13]. The moderate form of the disease has similar symptoms but is more subtle. The mild form often remains undiagnosed. Symptoms are subtle including mild headache and fever (up to 38°C), with the hemorrhagic syndrome restricted to small petechia on mucosa and skin [14, 15].

NE is endemic in the republic of Tatarstan, Russia [16]. We have previously demonstrated that Puumala orthohantavirus (PUUV) is the primary cause of NE in Tatarstan [17]. It is believed that endothelial cells are the primary targets of PUUV, where the virus can replicate without a cytopathic effect [18]. This is supported by the lack of tissue damage commonly found in postmortem specimens [19]. Therefore, immune mechanisms have been suggested to play a key role in the pathogenesis of NE. We have previously shown activation of proinflammatory cytokines in the serum of NE patients [20], where the severity of the disease was associated with high levels of circulating TNF-*α* and IL-1*β*. We have also shown that the mild form of NE is characterized by increased serum levels of IFN-*γ* and IL-12 [21]. Our data corroborate the findings of several other groups demonstrating cytokine production by infiltrating immune cells in the kidneys rather than the kidneys themselves. Based on a large body of data, it is generally considered that the clinical symptoms of NE are the result of a “cytokine storm” in response to the virus [22, 23].

There are multiple evidence strands pointing to those cytokines playing a primary role in the pathogenesis of NE [20, 21, 24, 25]. Nevertheless, our knowledge of the role of cytokines in the severity of NE disease remains limited. Therefore, in the current work, we tested the hypothesis that patients with NE have a markedly different serum cytokine profile to healthy controls by screening both groups of subjects for serum concentrations of 48 cytokines associated with immune responses to infection and we link these responses to markers of pathology experienced by patients. Our findings support previous work in that a more extreme inflammatory cytokine profile was associated with markers of acute kidney injury and that this cytokine profile was more marked in younger patients.

## 2. Materials and Methods

### 2.1. Subjects

Serum samples were collected from 139 patients (117 males and 22 females) and controls 57 (21 males and 36 females). Clinical records (including clinical pathology records) were also collated for these patients. Additionally, clinical laboratory test results such as serum levels of potassium ion triglycerides, cholesterol, very low density cholesterol (VLDCL), low-density cholesterol (LDCL), and high-density cholesterol (HDCL), routinely done upon hospitalization, were collected. Data were collected during the acute (VLDCL1, LDCL1, and HDCL1) and convalescent (VLDCL2, LDCL2, and HDCL2) phases of HFRS. The diagnosis of HFRS was established based on clinical presentation and was serologically confirmed by the detection of anti-hantavirus antibodies. Samples were collected following the standard operating procedure protocol in the hospital for the diagnosis of hantavirus infection and stored at -80°C until used.

### 2.2. Ethics Statement

The ethics committee of the Kazan Federal University approved this study, and signed informed consent was obtained from each patient and controls according to the guidelines adopted under this protocol (protocol 4/09 of the meeting of the ethics committee of the KSMA dated September 26, 2019).

### 2.3. Hantavirus ELISA

The Hantagnost diagnostic ELISA kit (Institute of Poliomyelitis and Viral Encephalitis, Moscow, Russia) was used to determine hantavirus-specific antibody titers as per the manufacturer's instructions. Briefly, NE patient and control sera were diluted 1 : 100 (PBS) and incubated for 60 min at 37°C in a 96-well plate with preadsorbed hantavirus antigens. Following washes (3x; 0.5% Tween20 in PBS, PBS-T), wells were incubated with anti-human-IgG-HRP conjugated antibodies (1 : 10000 in PBS-T, Amerixan Qualex Technologies, USA) for 30 min at 37°C. Postincubation and washes (3x; 0.5% Tween20 in PBS), wells were incubated with 3,3′,5,5′ tetramethylbenzidine (Chema Medica, Moscow, Russia). The reaction was stopped by adding an equal amount of 10% phosphoric acid (TatKhimProduct, Kazan, Russia). Data were measured using a microplate reader Tecan 200 (Tecan, Switzerland) at OD_450_ with reference OD_650_. OD_450_ values higher than 0.5 were considered positive results.

### 2.4. Multiplex Analysis

Serum levels of 48 analytes were analyzed using Bio-Plex (Bio-Rad, Hercules, CA, USA) multiplex magnetic bead-based antibody detection kits following the manufacturer's instructions. Multiplex kits, Bio-Plex Pro Human Cytokine 21-plex, and Bio-Plex Human Cytokine 27-plex panels were used in the study. Serum aliquots (50 *μ*l) were analyzed where a minimum of 50 beads per analyte was acquired. Median fluorescence intensities were collected using a Luminex 100 or 200 analyzer (Luminex, Austin, TX, USA). Each sample was analyzed in triplicate, and the resulting data were analyzed with MasterPlex CT control software and MasterPlex QT analysis software (MiraiBio, San Bruno, CA, USA). Standard curves for each cytokine were generated using standards provided by the manufacturer. Data were analyzed using MasterPlex CT control software and MasterPlex QT analysis software (MiraiBio, Alameda, CA, USA).

### 2.5. Statistical Analysis

#### 2.5.1. Clinical Symptoms Analysis

Analysis of clinical symptoms (presence or absence of each symptom in turn) was by log linear model selection of contingency tables in IBM SPSS Statistics version 24, based on maximum likelihood. Initially, full factorial models comprising symptom (2 levels, presence/absence) x sex (2 levels, male/female) x age (two levels, ≤40/>40 years old) were fitted and then simplified by the backward selection procedure to generate minimum sufficient models (MSM) for which the likelihood ratio of *χ*^2^ was not significant, indicating that the model was sufficient in explaining the data. The importance of each individual term in MSMs was assessed by the probability that its exclusion would alter the model significantly, and relevant *χ*^2^ values with associated probabilities provided. Quantitative clinical data were analyzed by multivariate GLM models in R version 2.2.1 (R Core Development Team).

#### 2.5.2. Analysis of Individual Cytokines

Preliminary analysis of individual cytokines was done using the nonparametric Mann–Whitney test with Benjamini-Hochberg (BH) adjustment for multiple comparisons using R language for statistical computing (R Core Development Team). The threshold used for statistical significance was *P* < 0.05.

#### 2.5.3. Cytokine Analysis Using Principal Component Analysis (PCA)

Since the data comprised values for 48 different cytokines and their receptors, in order to avoid the risk of Type I and Type II statistical errors, we first conducted a PCA in IBM SPSS vs. 24. The major principal components (PCs) responsible for the majority of variance in the data were then subjected to statistical analysis via two generalized linear models (GLMs) in R version 2.2.1.

PC1 and PC2 did not conform to Gaussian distributions, and all attempts to fit models with normal error structures failed to generate normally distributed residuals. The best-fit distributions were negative binomial. Therefore, the data were transformed by the addition of 0.85 to PC1 values and 1.38 to PC2 values to convert all records to positive values, then multiplied by 100 to avoid decimals, and rounded off to the nearest integer. These values were then used in GLMs.

Summary data are presented as arithmetic means of the PC and standard errors of the mean (S.E.M.). We fitted models in R with PC1 or PC2 as the dependent variables. Each subject's age was fitted as a covariate. Sex (at two levels, males and females) and subject's status (at two levels, patient or control) were fitted as fixed explanatory factors. Full factorial models that converged satisfactorily were simplified using the backward selection procedure and tested for significance at each step using deletion of terms beginning with the highest order interaction by comparing models with or without that interaction (3-way interaction). This was followed by models based on main effects plus 2-way interactions, and deletion of 2-way interactions in turn, and so on until each main effect was evaluated in a model that only comprised all main effects. Models were evaluated by the likelihood ratio (LR) and associated probability of rejecting the null hypothesis. Minimum sufficient models (MSMs) were then fitted (all significant main effects and any significant interactions), and the process was repeated to obtain values for changes in 2 x log-likelihood, test statistic (likelihood ratio (LR)), and probabilities.

The acceptability of GLMs was evaluated through the goodness-of-fit of residuals from MSMs through Q-Q plots and through estimation of the total variance accounted for by the model. The percentage of variance accounted for by each significant main effect or interaction was calculated as recommended by Xu (2003) and reported earlier by Behnke et al. (2008) and more recently by Grzybek et al. (2015a).

Finally, we fitted a multivariate model in R in which we included PC1, PC2, age, and sex as explanatory factors and six markers of pathology that were available for both patients and controls, as the dependent variables. In order to illustrate how markers of pathology vary in relation to increasing values of PC1 and PC2, we divided the values of each into four ranges and that of the controls, as follows:

PC1:

(1) $$\begin{array}{c} \text{Control subjects range}=-0.827\text{ to}-0.367,\\ \text{Patients range }1=-0.703\text{ to}-0.369\;\left(\text{all within the control range},n=57\right),\\ \text{Patients range }2=-0.344\text{ to}+0.973\;\left(\text{marginally above control range},n=52\right),\\ \text{Patients range }3=+1.022\text{ to}+1.780\;\left(\text{much higher than control range},n=17\right),\\ \text{Patients range }4=+2.035\text{ to }4.195\;\left(\text{very much higher than control range},n=11\right). \end{array}$$

PC2:

(2) $$\begin{array}{c} \text{Control subjects range}=-0.723\text{ to}-0.070\;\left(\text{with one extreme exception at }0.548\right),\\ \text{Patients range }1=-1.352\text{ to}-0.086\;\left(\text{all within the control range},n=58\right),\\ \text{Patients range }2=-0.074\text{ to}+0.492\;\left(\text{marginally above control range},n=46\right),\\ \text{Patients range }3=+0.506\text{ to}+1.689\;\left(\text{much higher than control range},n=27\right),\\ \text{Patients range }4=+1.845\text{ to }7.401\;\left(\text{very much higher than control range},n=6\right). \end{array}$$

## 3. Results

### 3.1. Clinical Presentation of NE Cases

HFRS diagnosis was based on clinical presentation and epidemiological data as well as serological confirmation. The average hospitalization period was 9.4 ± 0.4 days and the average duration of the febrile period 6.8 ± 0.1 days. Clinical and demographic data are summarized in Table 1.

The clinical form of the disease was classified as mild, moderate, or severe. There were more male patients as compared to female diagnosed with NE. The mild form was characterized by fever (38°C), oliguria (900 ml/day; 39% of patients), micoproteinuria (0.1 g/l), a normal level of urea (1.7-8.3 mM/l), and increased levels of creatinine (up to 130 mkM/l). Hemorrhagic syndrome presented as nose bleeding in 5% of patients. Patients with the moderate form of HFRS had fever (39.5°C), headache, frequent vomiting and abdominal pain, back pain, multiple petechias, oliguria (300 ml/day; 68.6%), and levels of urea and creatinine up to 18 mM/l and 300 mkM/l, respectively. The moderate form of HFRS was characterized by pronounced hemorrhagic syndrome (10.2%), which included nose bleeding (8.8%) and petechias (5.8%). In contrast, patients with the severe form of HFRS had complications such as shock, acute cardiovascular insufficiency (22.5%), hemorrhages (74.1%), oliguria (less than 300 ml/day; 100%) or anuria (54.8%), and levels of urea and creatinine higher than 18.5 mM/l and 300 mM/l, respectively. In addition, 16.1% of patients required hemodialysis. Hemorrhagic syndrome in these patients included nose bleeding (67.7%), hemorrhages (38.7%), and scleral hemorrhages (25.8%).

Next, we sought to determine whether frequency of clinical symptoms differed depending on sex and age of NE (Table 2). As expected, the severity of symptoms worsened with the disease class (class 1: mild; class 2: moderate; and class 3: severe). We also found a higher frequency of hemorrhagic (nose bleeding and petechia) and gastrointestinal (diarrhea and abdominal) symptoms in male as compared to female patients. Additionally, symptoms of renal dysfunction (anuria and oliguria) as well as fog in eye were more often described in male as compared to female patients. Only one symptom, cough, was found more frequently in females as compared to male subjects.

In the case of fog eye, there were also two significant interactions: age x sex, *P* = 0.017 and sex x severity, *P* < 0.001.

We acknowledge that the number of samples in sex groups differ, having more male as compared to females, which is characteristic for NE [1, 26]. Therefore, this discrepancy in number of samples could be a factor affecting the analysis.

When NE symptoms were analyzed based on age of the patient, we found that younger patients (≤40 years old) had a higher frequency of hemorrhagic (petechia), gastrointestinal (vomiting, nausea, and abdominal pain), and eye fog symptoms as compared to older (>40 years old) NE. Also, younger patients presented with kidney dysfunction (anuria and oliguria) symptoms more often as compared to older NE. Cough was the only symptom which was more frequent in older as compared to younger NE patients. These data indicate that clinical presentation of NE depends on sex and age of the patient. Although multiple factors could contribute to variation of NE, activation of cytokines could play a substantial role.

### 3.2. Analysis of Cytokine Levels

The mean values of cytokine and receptor levels detected in the sera are given in Table 3, which also shows the arithmetic difference between values in patients and the control group, as well as the relative change in value between these groups (mean value of patients divided by that of controls). With the exception of IL-1*α* and CCL27, the mean levels of all the other cytokines were arithmetically higher in patients relative to controls.

Analysis was based on PCA to avoid statistical errors arising from multiple tests, as explained above (Materials and Methods). PCA identified in total 13 components as quantifiable (collectively accounting for 80% of variance). PC1 was the dominant component accounting for almost a quarter of total variance (23.1%), and PC2 explained the next 15.3%. Between them, therefore, these two accounted for 38% of the variance. None of the other PCs accounted for more than 7% of variance, and these were not studied further.

**Figure 1:**
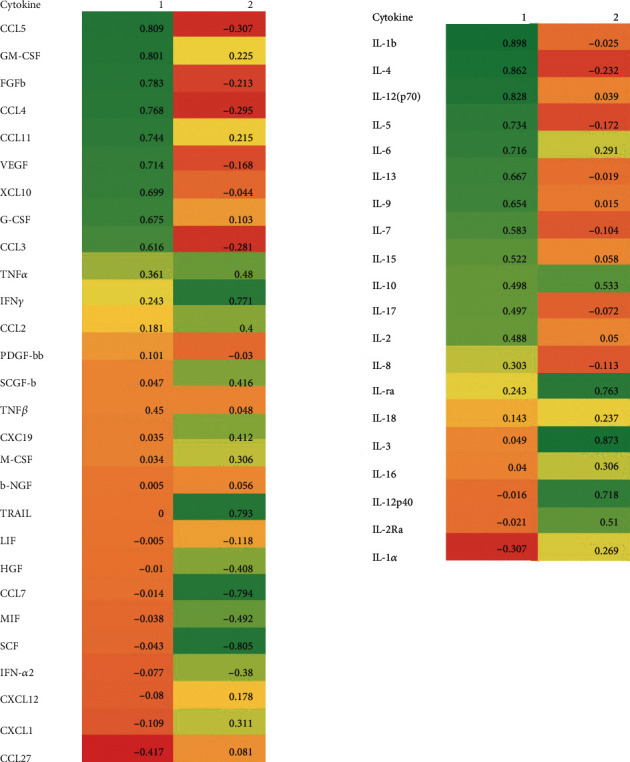
The proportional contribution of each cytokine/receptor to the two principal components: PC1 and PC2.

Twenty-eight of the cytokines and receptors contributed positively to PC1 (Figure 1), with values ranging from 0.898 to 0.101. The greatest positive contribution was from IL-1*β* (0.898), IL-4 (0.862), IL-12 (0.828), CCL5 (0.809), and GM-CSF (0.801). Three cytokines (CXCL1, IL-1*α*, and CCL27) made negative contributions to PC1 (-0.109, -0.307, and -0.417, respectively). Twenty-seven cytokines and receptors contributed positively to PC2, the greatest contributions being from IL-3 (0.873), SCF (0.805), CCL7 (0.794), TRAIL (0.793), IFN-*γ* (0.771), IL-1ra (0.763), and IL-12p40 (0.718). There were nine negative contributions greater than -0.1, as shown in Figure 1.

### 3.3. Frequency Distributions of PC1 and PC2

**Figure 2:**
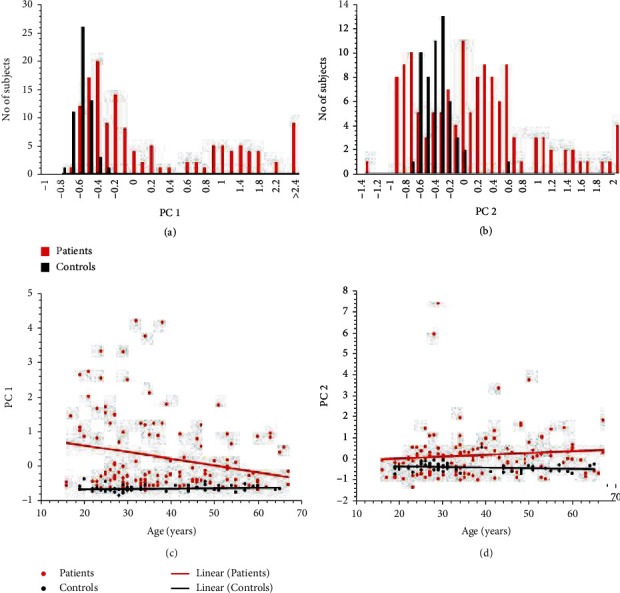
Frequency and age distribution of PC1 and PC2 in NE and controls. Frequency of PC1 (a) and PC2 (b) distributions in NE and controls. Frequency of PC1 (c) and PC2 (d) distributions based on age of NE and controls. For details of the statistical analysis, see the text.

The frequency distributions of PC1 and PC2 are illustrated in Figures 2(a) and 2(b), respectively. The values of PC1 in controls did not exceed -0.3, and 56 patients also had values in the control range (Figure 2(a)). The remaining patients had higher values, the first of which form an extension to the peak that includes controls, and then perhaps up to 2-3 peaks at higher values of PC1. These suggest different degrees of responsiveness to infection. The difference between patients and controls was highly significant (GLM with negative binomial errors, main effect of subject status, *LR*_1,189_ = 108.75, *P* < 0.0001), accounting for 5.22% of the variance in the data. Figure 2(b) shows that values of PC2 in controls, with just one exception, were restricted to values less than -0.06. Twenty-five patients had values in the control range and some even lower and, as with PC1, there appeared to be several clusters in patients at higher values. The difference between patients and controls was highly significant (GLM with negative binomial errors, main effect of subject status, *LR*_1,190_ = 26.378, *P* < 0.0001), accounting for 1.2% of the variance in the data.

### 3.4. Relationship of PC1 with PC2

**Figure 3:**
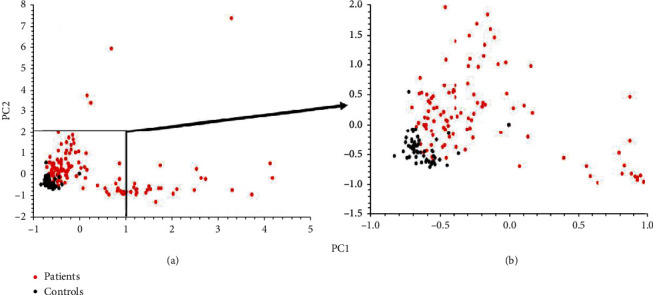
The relationship of PC1 to PC2 responses. Because of the tight clustering of control values with some overlapping values from patients in the bottom left hand corner of (a). This section is magnified in (b).

The relationship of PC1 to PC2 is shown in Figure 3, where it can be seen that values for control subjects cluster tightly in the bottom left-hand corner. This figure shows that many of the subjects with high PC1 values kept PC2 values in the control range, although some with relatively low PC1 values had high PC2 values, outside the control range. Moreover, there were just two patients with very high values for both. If we take the control values as -0.827 to -0.367 for PC1 and -0.723 to-0.076 for PC2, only 15 (10.8%) patients had PC1 and PC2 values that lie in this area on the figure, and therefore, 89.2% had increased serum levels of both the cytokines reflected in PC1 and PC2.

### 3.5. Age-Dependent Variation in PC1 and PC2

The mean value of PC1 in male (−0.642 ± 0.016) and female (−0.640 ± 0.017) controls was almost identical. Among patients, the mean value of PC1 was arithmetically higher in male subjects (0.306 ± 0.104) compared with females (−0.011 ± 0.177). However, the S.E.M.s are large, and therefore, with age taken into account, there was no overall significant difference between the sexes (GLM with negbin errors, main effect of sex, *LR*_1,188_ = 0.579, *P* = 0.447) and no significant interaction between subject status (patient or control) and sex (*LR*_1,185_ = 0.399, *P* = 0.528). Post hoc analysis by the Mann–Whitney *U* test confined to patients confirmed that PC1 did not differ between the sexes (*U*_116,21_ = 975.0, *P* = 0.147). Nevertheless, many of the high values for PC1 were from male subjects. In 95% of female subjects for which PC1 could be calculated, PC1 ranged from -0.656 to 0.947, and with only one exception of a female subject with a value of 2.547. In contrast, among male subjects, 28 subjects (24.1%) had values exceeding 0.947 and seven (6.0%) values exceeding 2.547.

The data in Figure 2(c) show that there is a tendency for younger patients to have high values of PC1, and with subject status taken into account, there was a significant effect of host age (GLM with negbin errors, subject status x age, *LR*_1,189_ = 7.524, *P* = 0.0061) accounting for 0.379% of the variance in the data. As patients aged, their PC1 values decreased (*β* = −0.02, *R*^2^ = 0.058, *t* = −2.873, *P* = 0.005). However, among controls, there was a very subtle increase in PC1 values with age but this was not significant (*β* = 0.001, *R*^2^ = 0.025, *t* = 1.167, *P* = 0.248). These different slopes in the relationship between age and PC1 values generated a significant 2-way interaction (GLM with negbin errors, subject status x age, *LR*_1,188_ = 6.136, *P* = 0.0132) accounting for 0.311% of the variance in the data.

For PC2, the values in control subjects were also very similar in the two sexes (males = −0.422 ± 0.037, females = −0.410 ± 0.041). Although this time the values were arithmetically higher for female patients (0.278 ± 0.211) compared with males (0.146 ± 0.108), the difference between the sexes was not significant (GLM with negbin errors, main effect of sex, *LR*_1,188_ = 0.129, *P* = 0.719), nor was the 2-way interaction significant (subject status x sex, *LR*_1,185_ < 0.001, *P* = 0.993). Post hoc analysis by the Mann–Whitney *U* test confined to patients confirmed that PC2 did not differ between the sexes (*U*_116,21_ = 1397.0, *P* = 0.285).

The age-distribution of PC2 is illustrated in Figure 2(d). Neither the main effect of age (*LR*_1,188_ = 0.992, *P* = 0.319) nor the 2-way interaction, age x subject status (*LR*_1,185_ = 1.500, *P* = 0.221) were significant in the case of PC2. The slope for patients is *β* = 0.009 (*R*^2^ = 0.010, *t* = 1.160, *P* = 0.248) and that for the controls *β* = −0.003 (*R*^2^ = 0.033, *t* = −1.338, *P* = 0.187). Two huge outliers can also be seen in Figure 2(d), presumably subjects that have overreacted.

### 3.6. Age-Dependent Variation in Specific Cytokines

**Figure 4:**
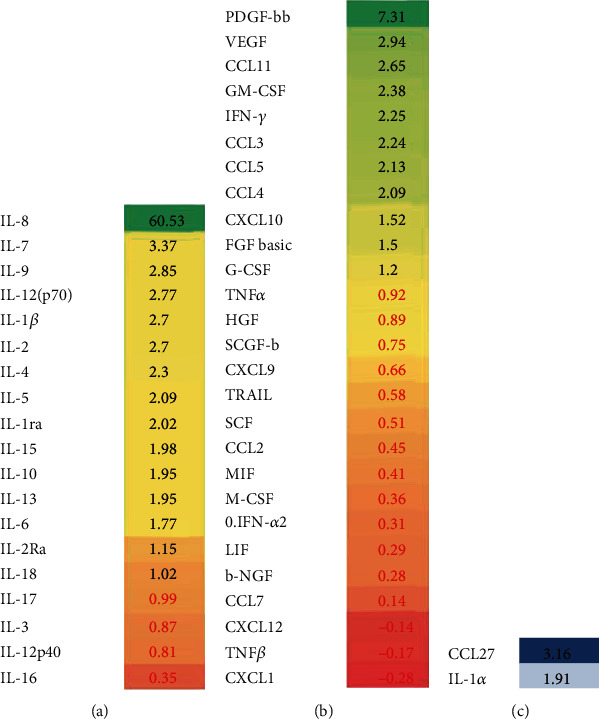
Heat map analysis of serum cytokine difference based on age of NE patient.

To examine how individual cytokine levels differ between age classes, we separated patients into two groups: younger (≤40 years old) and older (>40 years old) (Figure 4; Table S1). The relative response of each age class to their respective controls was calculated from the ratio of these responses (i.e., mean values in age class 1 (patients minus controls) divided by mean value in age class 2, (patients minus controls), and these are illustrated in the form of a heat map in Figure 4). The majority of cytokines were upregulated in both groups of patients as compared to controls (positive values in Table S1; column: arithmetic difference), suggesting that pathogenesis of the disease was mainly similar in both groups. Post hoc Mann–Whitney analysis revealed that 43 cytokines differed significantly between NE and controls in the younger age class, while among older subjects 41 differed.

Among the resulting ratios twenty six cytokines were higher, while twenty two cytokines were lower in younger as compared to older NE (Table S1; column: X difference). One cytokine in particular, IL-8, had a particularly high value indicating that young male subjects responded much more intensively compared to their age matched controls, than did older subjects (in older subjects the mean levels of IL-8 were only marginally higher than those of their age-matched controls). However, there were three cytokines (CXCL1, CXCL12, and TNF*β*), which were lower in the sera of younger patients as compared to their age-matched controls, while in older patients the levels of these cytokines were higher than among their respective controls. Of note, only two cytokines, IL-1*α* and CCL27, were lower in both age classes relative in each case to their age-matched controls. Post hoc analysis using Mann–Whitney *U* test identified three cytokines which were significantly higher in younger as compared to older NE (Table S1).

### 3.7. The Relationship of PC1 and PC2 to Measures of Pathology

We fitted a multivariate model in R, with six measures of pathology as the dependent variables. In the first run of this model sex was not a significant factor (Pillai trace statistic = 0.043, *F*_6,167_ = 1.24, *P* = 0.287). Therefore, sex was removed from the model, and all remaining explanatory factors retained significance. The strongest effect was from PC1 (Pillai trace statistic = 0.233, *F*_6,169_ = 8.53, *P* < 0.0001). Age (Pillai trace statistic = 0.089, *F*_6,167_ = 2.74, *P* = 0.014) and PC2 (Pillai trace statistic = 0.076, *F*_6,167_ = 2.31, *P* = 0.036) had weaker effects on the six dependent variables (the six measures of pathology).

**Figure 5:**
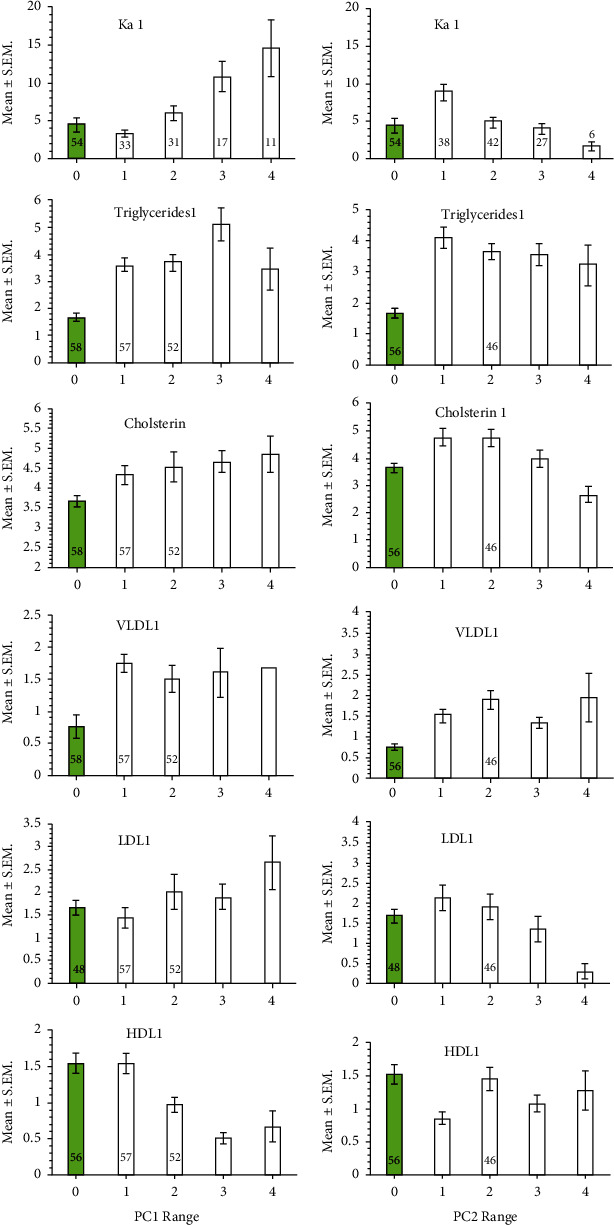
Measures of pathology in control subjects (green columns, labelled 0) and in patients (white columns, labelled 1-4), according to four ranges of PC1 and PC2. The sample sizes are given in respective columns in the top two panels, while in those below only if they differed from those in the top panel. Ka − 1 = potassium.

In order to illustrate these effects of PC1 and PC2 on measures of pathology, each PC was divided into four ranges and plotted alongside the values from control subjects (Figure 5). Thus, with age and subject status (patient and control) taken into consideration, for potassium levels, the effect of PC1 was positive and significant (*β* = 3.284, *t* = 6.605, *P* < 0.0001), while that of PC2 was negative and significant (*β* = −1.259, *t* = −2.738, *P* = 0.0068). The levels of triglycerides did not vary significantly with PC1 or PC2 despite the higher means when age and subject status had been controlled for. Cholesterol levels did not vary significantly with PC1 but showed significant negative decline with increasing values of PC2 (*β* = −0.375, *t* = −2.728, *P* = 0.0070). Neither PC1 nor PC2 affected the levels of VLDCL1 significantly. The levels of LDCL1 varied positively with increasing PC1 (*β* = 0.402, *t* = 2.710, *P* = 0.0074) and negatively with increasing values of PC2 (*β* = −0.334, *t* = −2.43, *P* = 0.0160), while those of HDCL1 fell significantly with increasing values of PC1 (*β* = −0.252, *t* = −4.402, *P* = 0.0001) but did not vary significantly with PC2.

## 4. Discussion

Cytokines play an important role in the pathogenesis of NE [20, 21]. We have previously demonstrated upregulation of proinflammatory cytokines in NE patients, including increased levels of CXCL8 and IL-10 as compared to controls [21]. Previously, we have shown also that serum TNF*α* and IL-1*β* were upregulated in severe HFRS [20] and we have demonstrated that levels of IL-6, CXCL10, CCL2, and CCL3 are associated with clinical presentation of the disease. In this earlier study, the serum level of only a limited number of cytokines was analyzed. Therefore, building on our previous work, in the current analysis, we included 48 cytokines and receptors, including leukocytes, chemokines, growth factors, and interferons and proinflammatory cytokines. We found marked changes in the levels of a large number of cytokines especially in subjects with the severe form of NE as compared to mild and moderate forms of the disease at the febrile stage of the disease.

The results here demonstrate that the cytokine profile does indeed vary with disease with a proinflammatory profile (PC1) being associated with several markers of acute kidney injury (hyperkalemia, oliguria, elevated creatinine and perturbations in cholesterol ratio). This proinflammatory profile was more marked in younger patients, a finding that is concordant with the known overrepresentation of younger patients in those with clinical disease and the known higher prevalence of hantavirus infection in younger compared with older patients. [27–29]. It has been suggested that “cytokine storm” best explains the pathogenesis of hantavirus infection [22, 25]; however, little is known about how serum cytokine levels vary with host age. NE is diagnosed in patients of all ages [16, 30]; however, it appears that recovery is more prolonged in young female patients [31], and young male patients have a higher risk of developing serious complications of the central nervous system [27]. The mechanisms underlying these serious consequences remain largely unknown but our findings of an association between proinflammatory cytokines and the young age of patients could provide an explanation. This activation of the proinflammatory profile fits the “cytokine storm” model, where strong activation of cytokines is linked to tissue damage and, potentially a fatal outcome [32]. Multiple cytokines and chemokines, such as IL-1*β*, IL-6, CXCL10, CCL2, CCL11, G-CSF, and GM-CSF, have been shown to be associated with cytokine storms [33]. These cytokines we found upregulated in young patients (Supplemental Table 1), suggesting their contribution to the pathogenesis of the disease in this NE subset of the study group.

A high male to female ratio in the disease has been demonstrated in multiple studies [16, 29, 34]. Krautkramer et al. suggested that a higher risk of exposure among male compared to female subjects may explain the male bias in NE diagnoses [35]. In another study, the difference between male and female subjects in the risk of contracting hantavirus infection was hypothesized to be attributable to sex-related differences in expression of various estrogen receptors [36]. The role of cytokines in sex-associated pathogenesis of hantavirus infection has been demonstrated by Klingstrom et al. where high levels of IL-8 and CXCL10 were identified in male as compared to female NE [23]. Our results concur with the results of this study in that we also found that the levels of IL-8 and CXCL10 in NE differ between the sexes. One of the most intriguing findings in our study was a substantial increase in IL-8 level in the serum of younger as compared to older NE patients. This cytokine is a potent chemokine, attracting neutrophils to the site of infection [37] and favors the formation of neutrophil extracellular traps [38]. IL-8-exposed neutrophils have higher adhesion to endothelial cells [39], transendothelial migration [40], and tissue damage [41]. IL-8 may cause tissue damage by releasing matrix metalloproteases degrading extracellular matrix components [42]. Supporting the pathogenic role of IL-8 in NE is data presented by Strandin et al., where a positive correlation between the serum level of this cytokine and kidney dysfunction was demonstrated [43]. Increased serum levels of IL-8 in NE were shown also by Sadeghi et al. [44]. These authors demonstrated that cytokine serum levels were positively correlated with creatinine and C reactive protein, indicators of kidney dysfunction and inflammation. Our data expand understanding of the role of IL-8 in NE pathogenesis by identifying that younger patients respond most intensively with this cytokine. Therefore, we suggest that IL-8 may contribute to variation in clinical presentation in these groups of patients.

In agreement with Klingstrom et al. [23], we found also that younger males had higher levels of CXCL10 as compared to the same age group females (4330 vs. 179, respectively). Male subjects of both age classes had higher values than their respective age-matched controls (138.2 times higher than age-matched controls for younger males and 35.51 for the older males), while the younger females did not respond as well with his cytokine (only 3.5 times higher than age-matched controls). In contrast, the older females responded almost as well as the males (72.5 times higher than age-matched controls). It should be noted that the sex groups were unequal, with more female as compared to male NE included. This is characteristic for NE as it is diagnosed more often in male as compared to female subjects [1, 26]. Therefore, this discrepancy in the number of samples could be a factor affecting the analysis. More samples from female NE in future studies will strengthen the robustness of analyses and resulting conclusions as to the role of sex in disease pathogenesis.

Although the levels of many of the cytokines that we measured were arithmetically higher in male as compared to female NE, our study did not reveal overall a significant difference in PC1 and PC2 between the sexes. The overriding importance of age in the cytokine profiles likely masks the complex interactions of host sex and age. A greater tendency towards a PC1 profile was demonstrated in male patients in this study with a more detailed scrutiny of individual cytokines indicating that the responses of young men and women differed in many cases to older patients of the same sex. While this study was of a reasonable size, it is likely that much larger age and sex matched cohort studies will be necessary to fully characterize these differences. Future studies would also need to take into account likely confounding factors such as the pre- and postmenopausal status of female patients in their cytokine responses.

Aging has profound effects on the functioning of the immune system. Declining antibody production is well documented in elderly populations [45], supporting the overall impaired response typical of this subset of the population. Some of the more striking differences are associated with reductions in T cell function and lowered IL-2 production [46, 47]. Lower IL-2 production in older as compared to younger NE patients was evident in our study (Figure 3; Supplemental Table 3). Also, five common *γ* chain cytokine family members (IL-2, IL-4, IL-7, IL-9, and IL-15) were found upregulated in younger patients (Figure 4; Table S2). As these cytokines play a pivotal role in the development, survival, proliferation and differentiation of the innate and adaptive immune responses [48], the lower level of these cytokines in older NE patients could contribute to disease pathogenesis in this cohort of patients.

It should be pointed out that genetic factors could contribute to age dependent differences in NE severity. Genetic mechanisms have been suggested also to play role in cytokine storms, the leading factor in pathogenesis of hantavirus infection [25, 49, 50]. Recent studies of genetic factors have implicated several *IL6* gene variations in pathogenesis of coronavirus infection 2019 (COVID-19) [51], a disease where severity has a strong association with the likelihood of a cytokine storm [52]. Severity of influenza, another disease with cytokine storm-based pathogenesis has been associated also with *IL1B* gene polymorphism [53]. The contribution of genetic factors to pathogenesis of hantavirus infection has been investigated also [54]. Multiple human leukocyte antigen alleles (HLA) have been shown as connected to the severe form of infection [55, 56]. Additionally, a haplotype associated with high production of TNF-*α* has been correlated with the severe form of NE [57]. Also, *IL-1RA* allele 2 and *IL-1b* allele 2 have been found to be less frequent in hantavirus-infected patients as compared to seronegative controls [58]. The contribution of these genetic factors to pathogenesis of NE could be modified by age, environment, and ethnicity [59–61].

We found some associations between biochemical laboratory data and cytokine PCs; notably, the serum potassium levels (a marker of acute kidney injury) positively correlated with proinflammatory PC1 cytokines. Interestingly, IL-1*β*, a major proinflammatory cytokine, has been shown to inhibit the inwardly rectifying K+ channel in human proximal tubule cells [62, 63]. This could avert the intake of potassium leading to accumulation of this ion in the interstitial space and in the serum. In the kidneys, IL-1*β* causes suppression of K+ channels which could lead to lower reabsorption of Na+ [64] and glucose [64], contributing to oliguria, the main symptom of NE [14]. Interestingly, glucosurea is detected in PUUV-infected patients and it has been shown to correlate with disease severity [65]. Therefore, it could be suggested that the markers of NE severity could be the result of the effects of proinflammatory cytokines on kidney cell potassium transport function.

We found also that LDCL1 and HDCL1 have positive and negative associations with PC1 cytokines, respectively. Changes in the serum level of lipids have been demonstrated in hantavirus-infected patients [2, 66, 67]. Our results provide more data contributing to the understanding of the role of lipids in pathogenesis of NE. The association between LDL and proinflammatory cytokines has been demonstrated in multiple studies, where IL-1 and TNF*α*, the main contributors to “cytokine storms” in model organisms or pathology, were shown to increase plasma low-density lipids [68, 69]. In turn, low-density lipids can activate production of IL-1*β* and IL-18 by engaging Toll-like receptors (TLRs) and triggering the formation of inflammasomes [70]. In contrast, HDL was shown to have anti-inflammatory effects by reducing expression of TLRs and reduced IFN receptor signaling [71]. Our data also support the notion that HDL could have an anti-inflammatory effect as a negative association was found in NE between HDL and PC1 cytokines. These data suggest that serum LDL and HDL could contribute to the pathogenesis of NE; however, the mechanisms remain to be determined.

## 5. Conclusion

NE is an acute zoonotic disease which is characterized by kidney insufficiency and hemorrhages. Although diagnosed in both sexes, higher male to female ratios in NE are often reported [35]. The pathogenesis of the disease remains largely unknown; however, excessive cytokine activation, known as “cytokine storm,” is suggested to play a role. Finally, we identified that high serum levels of potassium and LDL were associated with PC1 cytokines, while serum HDL had an opposite association with the proinflammatory cytokine profile. These associations between the PC1 cytokine profile and HDL, as well as LDL, are recorded for the first time. Our data suggest an important role for proinflammatory cytokines in the pathogenesis of NE, especially, in young patients.

## Figures and Tables

**Table 1:** Demographic, clinical, and laboratory information for NE.

Variables	Value
Age (years)	38 ± 12.9
Sex (M/F)	117/22
Age M (years)	38.4 ± 12
Age F (years)	47.4 ± 14
Mild form HFRS (%)	10.07
Moderate form HFRS (%)	58.23
Severe form HFRS (%)	23.72
Mild HFRS M/F	17/8 (14.5%/36.4%)
Moderate HFRS M/F	71/11 (60.7%/50%)
Severe HFRS M/F	29/3 (24.8%/13.6%)
Antibody titer (1^st^)	1 : 200
Antibody titer (2^nd^)	1 : 800
Hospitalization (days)	9.4 ± 4.7

**Table 2:** Prevalence of clinical symptoms according to severity of disease and age.

Symptom	Symptom class	Symptom severity prevalence (CL_95_)	Sex	Sex prevalence (CL_95_)	Age class	Age prevalence (CL_95_)
Nose bleed	1	4.0 (0.21-19.56)	Male	16.2 (11.35-22.47)	1	17.2 (9.27-29.07)
	2	9.9 (4.44-19.94)	Female	0.0 (0.0-15.17)	2	7.7 (3.59-15.03)
	3	30.3 (18.62-44.92)				
		^∗^		^∗^		NS
Petechia	1	0.0 (0.0-13.36)	Male	18.8 (13.51-25.42)	1	23.0 (13.63-35.72)
	2	4.9 (1.47-13.63)	Female	4.5 (0.24-22.21)	2	5.8 (2.46-12.31)
	3	57.6 (42.87-71.27)				
		^∗∗∗^		NS		^∗∗^
Scleral bleed	1	0.0 (0.0-13.36)	Male	6.8 (3.89-11.63)	1	6.9 (2.45-16.61)
	2	2.5 (0.38-10.00)	Female	0.0 (0.0-15.17)	2	3.8 (1.27-9.77)
	3	18.2 (9.31-31.91)				
		^∗∗^		NS		NS
Bleeding	1	4.0 (0.21-19.56)	Male	29.1 (22.67-36.33)	1	32.2 (21.14-45.12)
	2	17.3 (9.56-28.64)	Female	4.5 (0.24-22.21)	2	13.5 (7.73-21.77)
	3	60.0 (45.93-74.10)				
		^∗∗∗^		^∗∗^		NS
Cough	1	28.0 (13.37-47.97)	Male	5.1 (2.66-9.50)	1	3.4 (0.69-11.86)
	2	7.4 (2.85-16.76)	Female	31.8 (15.18-54.65)	2	19.2 (12.41-28.34)
	3	0.0 (0.0-8.04)				
		^∗∗∗^		^∗∗^		^∗^
Diarrhea	1	12.0 (3.36-30.31)	Male	35.0 (28.14-42.53)	1	35.6 (24.26-48.58)
	2	32.1 (21.46-44.55)	Female	31.8 (15.18-54.65)	2	23.1 (15.34-32.69)
	3	42.2 (28.73-57.13)				
		NS		^∗∗^		NS
Vomiting	1	8.0 (1.45-25.59)	Male	35.0 (28.14-42.53)	1	41.4 (29.15-54.36)
	2	34.6 (23.60-47.03)	Female	31.8 (15.18-54.65)	2	23.1 (15.34-32.69)
	3	54.5 (39.81-68.37)				
		^∗∗^		NS		^∗^
Nausea	1	36.0 (19.57-56.08)	Male	57.3 (49.76-64.51)	1	41.4 (29.15-54.36)
	2	48.1 (35.63-60.69)	Female	31.8 (15.18-54.65)	2	23.1 (15.34-32.69)
	3	78.8 (64.27-88.59)				
		^∗∗^		NS		^∗^
Abdominal pain	1	28.0 (13.37-47.97)	Male	67.5 (60.08-74.20)	1	70.1 (57.20-80.62)
	2	59.3 (46.76-71.06)	Female	27.3 (12.61-50.00)	2	46.2 (36.45-56.34)
	3	90.0 (78.91-96.71)				
		^∗∗∗^		^∗∗^		^∗^
Back pain	1	44.0 (25.60-64-25)	Male	65.8 (58.32-72.55)	1	69.0 (56.05-79.75)
	2	63.0 (50.48-74.10)	Female	59.1 (38.26-77.78)	2	57.7 (47.53-67.39)
	3	84.8 (71.35-93.03)				
		^∗∗^		NS		NS
Anuria	1	0.0 (0.0-13.36)	Male	14.5 (9.91-20.59)	1	17.2 [9.27-29.07)
	2	0.0 (0.0-6.07)	Female	4.5 (0.24-22.21)	2	5.8 (2.46-12.31)
	3	54.5 (39.81-68.37)				
		^∗∗∗^		^∗∗∗^		^∗∗∗^
Oliguria	1	20.0 (8.23-39.84)	Male	72.6 (65.46-78.85)	1	75.9 [63.12-85.47)
	2	70.4 (57.93-80.52)	Female	45.5 (26.05-66.17)	2	55.8 (45.60-65.46)
	3	100.0(91.96-100.0)				
		^∗^		NS		^∗^
Fog eye	1	16.0 (5.66-35.74)	Male	54.7 (47.19-62.00)	1	59.8 (46.79-72.00)
	2	44.4 (32.65-56.96)	Female	13.6 (3.83-33.82)	2	28.8 (20.64-38.90)
	3	81.8 (68.09-90.69)				
		^∗∗∗^		^∗∗∗^		^∗∗∗^

^∗^ *P* = 0.05 − 0.01, ^∗∗^*P* = 0.099 − 0.001, and ^∗∗∗^*P* < 0.001. For severity classes: 1, mild; 2, moderate; and 3, severe. The sample sizes for each class were 25, 81, and 33, respectively. Number of male patients = 117 and females = 22. Number of patients for age classes 1 (≤40 years old) and 2 (>40 years old) were 87 and 52, respectively. Prevalence is the percentage (%) of subjects showing the symptom in the relevant data subset. Cl_95_ are the 95% confidence limits. For further details, see text.

**Table 3:** Mean values (±S.E.M.) for all cytokines and receptors and the arithmetic difference between the mean values of patients and control subjects, in order of the magnitude of the change.

	Patients	Controls	Mean difference	X change	Mann–Whitney *U* test
	(*n* = 139)	(*n* = 57)	Patients minus controls	Patients/controls	*P* value
IL-1*α*	0 62 ± 0.08	1.292 ± 0.12	*-0.67*	*0.48*	0.0001^∗^
CCL27	69.89 ± 7.03	125.19 ± 10.00	*-55.303*	*0.56*	0.0001^∗^
CXCL12	45.85 ± 9.41	36.284 ± 6.14	9.563	**1.26**	0.38318
CXCL1	65.07 ± 6.034	51.481 ± 4.88	13.585	**1.26**	0.66127
CCL7	29.12 ± 2.75	18.724 ± 3.04	10. 394	**1.56**	0.02323^∗^
IL-8	63.04 ± 12.26	37.918 ± 15.57	25.123	**1.66**	0.00011^∗^
IL-16	215.12 ± 34.78	122.418 ± 9.91	92.703	**1.76**	0.04059^∗^
TNF*β*	2.23 ± 1.16	1.196 ± 0.27	1.035	**1.87**	0.23723
SCF	72.24 ± 7.04	33.774 ± 2.39	38.464	**2.14**	0.00066^∗^
IFN-*α*2	21.574 ± 3.40	8.958 ± 0.90∗	12.616	**2.41**	0.00012^∗^
TRAIL	43.553 ± 4.61	16.397 ± 2.52	27.156	**2.66**	0.00002^∗^
IL-3	201.265 ± 26.20	66.724 ± 5.96	134.541	**3.02**	0.00015^∗^
IFN-*γ*	100.881 ± 15.60	32.643 ± 4.32	68.238	**3.09**	0.0001^∗^
IL-18	27.441 ± 3.10	8.631 ± 1.48	18.78	**3.18**	0.0001^∗^
IL- 12p40	288.664 ± 32.77	88.116 ± 12.51	200.548	**3.28**	0.0001^∗^
MIF	518.034 ± 65.86	145.137 ± 25.43	372.897	**3.57**	0.0001^∗^
LIF	8.739 ± 2.68	2.404 ± 0.44∗	6.335	**3.64**	0.00004^∗^
M-CSF	5.809 ± 2.02	1.491 ± 0.14	4.318	**3.90**	0.0001^∗^
G-CSF	32.999 ± 2.22	8.074 ± 0.92	24.925	**4.09**	0.0001^∗^
HGF	402.173 ± 37.50	97.191 ± 13.56	304.982	**4.14**	0.0001^∗^
IL-1 ra	141.589 ± 30.78	31.442 ± 5.42	110.147	**4.50**	0.0001^∗^
IL-2ra	133.927 ± 15.07	28.862 ± 3.24	105.065	**4.64**	0.0001^∗^
SCGF - b	8486.585 ± 868.14	1564.75 ± 242.42	6921.838	5.42	0.0001^∗^
CCL11	89.706 ± 10.47	15.50 ± 2.90	74.21	5.79	0.0001^∗^
CCL2	89.357 ± 25.09	13.02 ± 1.33	76.34	6.85	0.0001^∗^
IL-7	14.519 ± 3.14	2.08 ± 0.40	12.44	6.98	0.0001^∗^
IL-5	8.067 ± 1.19	1.02 ± 0.24	7.043	7.88	0.0001^∗^
GM-CSF	23.25 ± 4.03	2.58 ± 0.69	20.67	9.01	0.0001^∗^
IL-15	53.60 ± 13.06	5.40 ± 0.86	48.196	9.92	0.0001^∗^
IL-12(p70)	38.73 ± 5.5	3.73 ± 0.48	35	***10.38***	0.0001^∗^
TNF-*α*	43.66 ± 9.10	4.17 ± 0.76	39.495	***14.48***	0.0001^∗^
VEGF	175.55 ± 25.20	15.15 ± 2.45	160.402	***11.59***	0.0001^∗^
*β*-NGF	8.54 ± 3.96	0.73 ± 0.06	7.809	***11.67***	0.0001^∗^
IL-6	39.42 ± 5.95	2.90 ± 0.65	36.516	***13.58***	0.0001^∗^
CXCL9	1797.09 ± 253.08	124.22 ± 18.93	1672.868	***14.47***	0.0001^∗^
FGF b	19.53 ± 2.12	1.29 ± 0.31	18.233	***15.10***	0.0001^∗^
IL-2	29.14 ± 9.54	1.78 ± 0.30	27.357	***16.33***	0.0001^∗^
IL-10	58.92 ± 11.58	3.56 ± 0.65	55.361	***16.55***	0.0001^∗^
IL-4	19.01 ± 2.54	1.10 ± 0.09	17.906	***17 16***	0.0001^∗^
IL-17	42.65 ± 8.87	2.27 ± 0.56	40.376	***18.76***	0.0001^∗^
IL-1*β*	15.81 ± 2.07	0.81 ± 0.15	15.007	***19.60***	0.0001^∗^
IL-9	96.2 ± 22.78	3.50 ± 0.51	92.698	***27.47***	0.0001^∗^
IL-13	36.93 ± 5.71	1 34 ± 0.13	35.588	***27.60***	0.0001^∗^
CCL3	47.83 ± 8.62	0.97 ± 0.34	46.857	***49.31***	0.0001^∗^
CCL5	3062.01 ± 398.29	60.99 ± 8.72	3001.024	***50.21***	0.0001^∗^
PDGF-bb	8105.64 ± 5756.86	144.56 ± 23.81	7961.073	***56.07***	0.0001^∗^
CXCL10	3497.04 ± 390.01	49.05 ± 7.17	3447.989	***71.29***	0.0001^∗^
CCL4	1020.56 ± 144.66	10.27 ± 2.13	1010.289	***99.37***	0.0001^∗^

*n*: numbers of control subjects in this case is 56. Mean difference: the arithmetic difference between the mean level of each cytokine in patients and controls (patient value minus control value). Numbers in italic are negative values indicating that the level of the cytokine was higher in controls relative to patients. Those in black show cytokine levels higher in patients compared to controls. X change: the ratio of the mean value in patients and that in controls (patient value divided by the control value). Here, numbers in italic have values less than 1, indicating that the level of the cytokine in each case was lower in patients than in controls. Numbers in bold show cytokine levels > 1 to 5 times higher in patients relative to controls. Numbers in black show cytokine levels > 5 to 10 times higher in patients relative to controls and those in bold italic show cytokine levels > 10 times higher in patients relative to controls. ^∗^Significantly different cytokines between NE and controls, *P* < 0.05, Mann–Whitney *U* test *P* < 0.05.

## Data Availability

The presented data (all data in the manuscript) used to support the findings of this study are included within the article.

## Supplementary Materials

Supplementary Materials Supplemental Table 1: analysis of serum cytokines based on sex of NE. Supplemental Table 2: analysis of serum cytokines in younger (≤40 years old) and older (>40 years old) NE. Supplemental Table 3: analysis of serum cytokines in younger (≤40 years old) and older (>40 years old) NE male. Supplemental Table 4: analysis of serum cytokines younger (≤40 years old) and older (>40 years old) NE female.
