# Supplementary material for: Serum Cytokine Alterations Associated with Age of Patients with Nephropathia Epidemica
Source: Biomed Res Int. 2022 Jan 11;2022:4685288. doi: 10.1155/2022/4685288 (PMC8766188; doi:10.1155/2022/4685288)
Supplement: Supplementary Materials — Supplemental Table 1: analysis of serum cytokines based on sex of NE. Supplemental Table 2: analysis of serum cytokines in younger (≤40 years old) and older (>40 years old) NE. Supplemental Table 3: analysis of serum cytokines in younger (≤40 years old) and older (>40 years old) NE male. Supplemental Table 4: analysis of serum cytokines younger (≤40 years old) and older (>40 years old) NE female. [file 4685288.f1.docx]

Supplemental Table 1. Analysis of serum cytokines based on sex of NE.

| **Cytokine** | **Male patients (n=117)** | **Female patients (n=22)** | **Male controls (n=21)** | **Female controls (n=36)** | **Male combined status (n=138)** | **Female combined status (n=58)** | **Difference (males - female)*** | | | |
| --- | --- | --- | --- | --- | --- | --- | --- | --- | --- | --- |
|  |  |  |  |  |  |  | Patients | Controls | Combined | |
| IL-1β | 17.164±2.38 | 8.63±3.04 | 0.64±0.16 | 0.91±0.22 | 14.65±2.07 | 3.84±1.25 | 8.531 | -0.269 | | 10.812 |
| IL-1ra | 156.012±36.37 | 64.89±12.14 | 30.32±8.58 | 32.10±7.07 | 136.88±31.08 | 44.54±6.64 | 91.124 | -1.783 | | 92.348 |
| IL-2 | 31.937±11.29 | 14.27±4.27 | 1.23±0.16 | 2.11±0.47 | 27.26±9.62 | 6.72±1.80 | 17.669 | -0.881 | | 20.543 |
| IL-4 | 20.08±2.89 | 13.35±4.48 | 1.15±0.12 | 1.08±0.12 | 17.20±2.52 | 5.74±1.85 | 6.733 | 0.064 | | 11.464 |
| IL-5 | 8.743±1.38 | 4.48±1.46 | 0.81±0.14 | 1.15±0.37 | 7.54±1.20 | 2.41±0.63 | 4.268 | -0.339 | | 5.125 |
| IL-6 | 40.804±6.67 | 32.12±12.67 | 2.39±0.42 | 3.20±1.00 | 34.92±5.76 | 14.17±5.13 | 8.689 | -0.809 | | 20.748 |
| IL-7 | 16.451±3.70 | 4.25±1.50 | 1.45±0.31 | 2.45±0.60 | 14.17±3.17 | 3.13±0.68 | 12.206 | -0.997 | | 11.039 |
| IL-8 | 69.474±14.44 | 28.83±7.24 | 19.07±4.58 | 48.91±24.45 | 61.80±12.35 | 41.29±15.39 | 40.649 | -29.847 | | 20.51 |
| IL-9 | 106.802±26.91 | 39.82±9.51 | 4.29±0.98 | 3.04±0.56 | 91.20±23.02 | 16.99±4.28 | 66.986 | 1.242 | | 74.21 |
| IL-10 | 65.841±13.64 | 22.13±5.49 | 3.57±0.69 | 3.56±0.96 | 56.37±11.71 | 10.60±2.45 | 43.715 | 0.017 | | 45.766 |
| IL-12(p70) | 40.431±5.99 | 29.70±14.01 | 3.07±0.64 | 4.12±0.66 | 34.75±5.21 | 13.82±5.51 | 10.736 | -1.042 | | 20.928 |
| IL-13 | 39.548±6.52 | 22.98±9.59 | 1.28±0.18 | 1.37±0.18 | 33.73±5.65 | 9.57±3.85 | 16.568 | -0.092 | | 24.157 |
| IL-15 | 54.157±15.19 | 50.63±17.63 | 5.89±1.51 | 5.12±1.05 | 46.81±12.95 | 22.38±7.24 | 3.531 | 0.767 | | 24.431 |
| IL-17 | 41.398±8.30 | 49.30±35.20 | 1.65±0.28 | 2.64±0.87 | 35.35±7.14 | 20.34±13.51 | -7.904 | -0.989 | | 15.011 |
| CCL11 | 92.711±10.60 | 73.73±35.19 | 13.48±3.96 | 16.67±3.99 | 80.65±9.32 | 38.31±13.88 | 18.986 | -3.191 | | 42.341 |
| FGF basic | 19.576±2.34 | 19.26±5.14 | 1.71±0.66 | 1.05±0.31 | 16.86±2.06 | 7.96±2.26 | 0.318 | 0.667 | | 8.903 |
| G-CSF | 33.239±2.31 | 31.73±6.94 | 8.42±1.19 | 7.87±1.28 | 29.46±2.11 | 16.92±3.12 | 1.513 | 0.547 | | 12.541 |
| GM-CSF | 25.49±4.69 | 11.34±4.49 | 3.55±1.59 | 2.01±0.59 | 22.15±4.04 | 5.55±1.82 | 14.149 | 1.541 | | 16.601 |
| IFN-γ | 108.67±18.21 | 59.48±16.23 | 36.39±7.77 | 30.46±5.18 | 97.67±15.63 | 41.47±7.11 | 49.191 | 5.929 | | 56.203 |
| CXCL10 | 3612.40±417.13 | 2883.52±1087.53 | 41.49±8.52 | 53.46±10.22 | 3069.00±370.03 | 1126.93±445.42 | 728.887 | -11.974 | | 1942.072 |
| CCL2 | 71.28±8.90 | 185.48±152.57 | 15.62±2.78 | 11.50±1.32 | 62.81±7.75 | 77.49±58.13 | -114.197 | 4.128 | | -14.676 |
| CCL3 | 51.76±9.88 | 26.93±13.83 | 0.55±0.15 | 1.22±0.53 | 43.96±8.52 | 10.97±5.44 | 24.827 | -0.669 | | 32.995 |
| PDGF-bb | 9365.82±6836.86 | 1403.75±575.87 | 167.84±51.31 | 130.99±23.33 | 7966.13±5799.55 | 613.76± | 7962.076 | 36.855 | | 7352.372 |
| CCL4 | 1092.68±164.66 | 637.00±252.97 | 12.08±5.05 | 9.22±1.70 | 928.24±143.40 | 247.34±230.74 | 455.685 | 2.86 | | 680.902 |
| CCL5 | 3359.83±448.61 | 1478.13±729.64 | 66.95±17.37 | 57.51±9.54 | 2858.74±393.30 | 596.37±287.69 | 1881.7 | 9.437 | | 2262.375 |
| TNFα | 40.90±8.93 | 58.35±32.91 | 4.55±1.42 | 3.94±0.89 | 35.37±7.65 | 24.58±12.80 | -17.446 | 0.616 | | 10.793 |
| VEGF | 186.01±27.87 | 119.94±57.96 | 13.67±4.13 | 16.01±3.07 | 159.78±24.21 | 55.43±22.75 | 66.075 | -2.349 | | 104.35 |
| IL-1α | 0.667±0.09 | 0.38±0.10 | 1.277±0.22 | 1.3±0.15 | 0.76±0.087 | 0.951±0.12 | 0.287 | -0.023 | | -0.191 |
| IL-2Ra | 128.468±16.737 | 162.961±33.99 | 35.702±7.08 | 24.873±2.93 | 114.351±14.50 | 77.251±15.60 | -34.493 | 10.829 | | 37.1 |
| IL-3 | 206.144±30.86 | 175.313±21.96 | 62.705±6.74 | 69.068±8.63 | 184.317±26.53 | 109.368±11.93 | 30.831 | -6.363 | | 74.949 |
| IL-12p40 | 292.859±38.26 | 266.352±39.36 | 82.26±15.64 | 91.532±17.72 | 260.811±33.14 | 157.843±21.50 | 26.507 | -9.272 | | 102.968 |
| IL-16 | 222.945±40.10 | 173.515±27.34 | 140.595±20.58 | 111.816±9.97 | 210.413±34.96 | 135.219±12.57 | 49.43 | 28.779 | | 75.194 |
| IL-18 | 24.41±2.21 | 43.369±15.54 | 7.668±2.48 | 9.193±1.86 | 21.863±1.97 | 22.156±6.32 | -18.959 | -1.525 | | -0.293 |
| CTACK | 64.409±7.07 | 99.022±23.14 | 121.497±15.83 | 127.344±13.01 | 73.096±6.67 | 116.601±11.95 | -34.613 | -5.847 | | -43.505 |
| CXCL12 | 37.659±6.60 | 89.391±47.84 | 31.295±8.10 | 39.193±8.54 | 36.691±5.72 | 58.23±18.92 | -51.732 | -7.898 | | -21.543 |
| HGF | 408.62±41.823 | 367.88±83.23 | 115.34±25.56 | 86.61±15.47 | 363.99±36.76 | 193.296±37.23 | 40.741 | 28.732 | | 170.695 |
| IFN-α2 | 18.95±1.545 | 35.55±19.97 | 8.14±1.18 | 9.45±1.26 | 17.30±1.37 | 19.524±7.82 | -16.602 | -1.316 | | -2.223 |
| LIF | 9.12±3.15 | 6.71±2.54 | 2.57±0.76 | 2.30±0.54 | 8.13±2.68 | 4.004±1.06 | 2.414 | 0.267 | | 4.121 |
| CCL7 | 29.13±3.1 | 29.07±5.62 | 19.33±4.83 | 18.37±3.95 | 27.64±2.74 | 22.429±3.30 | 0.054 | 0.965 | | 5.207 |
| M-CSF | 4.06±0.46 | 15.11±12.61 | 1.48±0.26 | 1.50±0.15 | 3.67±0.40 | 6.66±4.80 | -11.049 | -0.026 | | -2.996 |
| MIF | 521.54±70.63 | 499.42±183.00 | 175.62±50.6 | 127.33±27.58 | 468.90±61.25 | 268.47±74.44 | 22.115 | 48.346 | | 200.438 |
| CXCL9 | 1723.05±184.71 | 2190.86±1283.81 | 127.05±35.15 | 122.57±22.27 | 1480.18±164.07 | 907.10±498.18 | -467.807 | 4.48 | | 573.086 |
| b-NGF | 8.44±4.67 | 9.09±3.42 | 0.76±0.08 | 0.71±0.09 | 7.27±3.96 | 3.89±1.39 | -0.653 | 0.051 | | 3.379 |
| SCF | 65.30±6.52 | 109.14±27.01 | 33.57±3.21 | 33.89±3.33 | 60.47±5.63 | 62.43±11.38 | -43.838 | -0.323 | | -1.963 |
| SCGF-b | 9424.4±1007.61 | 3499.12±242.95 | 1267.59±256.42 | 1738.09±353.034 | 8183.15±890.48 | 2406.07±261.84 | 5925.284 | -470.5 | | 5777.081 |
| CXCL1 | 58.82±5.76 | 98.30±21.84 | 53.04±9.01 | 50.57±5.75 | 57.94±5.06 | 68.68±9.42 | -39.479 | 2.464 | | -10.737 |
| TNFβ | 0.80±0.076 | 10.19±7.54 | 0.90±0.17 | 1.37±0.42 | 0.82±0.07 | 4.62±2.81 | -9.388 | -0.47 | | -3.802 |
| TRAIL | 43.77±5.049 | 42.38±11.69 | 11.22±2.68 | 19.42±3.60 | 38.82±4.41 | 28.13±5.12 | 1.399 | -8.204 | | 10.693 |

- With reference to the final 3 columns

Green color – positive values here indicate that the mean cytokine level is higher in male compared with female subjects

Black color – negative values here indicate that the mean cytokine level is lower in male compared with female subjects

Supplemental table 2. Analysis of serum cytokines in younger (≤40 years old) and older (>40 years old) NE.

| **Cytokine** | **Patients (n=87)** | **Patients (n=52)** | **Controls (n=34)** | **Controls (n=23)** | **Arithmetic difference X difference** | | |
| --- | --- | --- | --- | --- | --- | --- | --- |
|  | **younger (40years and less)** | **older (>40 years)** | **younger (40 years and less)** | **older 2 (>40 years)** | **Patients - controls** | |  |
|  |  |  |  |  | **younger** | **older** | **younger/ older** |
| IL-1β | 20.2±3.05 | 8.48±1.74 | 0.55±0.17 | 1.2±0.26 | 19.66 | 7.28 | 2.7 |
| IL-1ra | 156.18±45.89 | 117.22±29.91 | 19.18±4.33 | 49.58±10.91 | 136.99 | 67.64 | 2.02 |
| IL-2 | 37.76±15.13 | 14.73±2.42 | 1.99±0.49 | 1.49±0.19 | 35.78 | 13.24 | 2.7 |
| IL-4 | 23.68±3.72 | 11.23±2.4 | 0.95±0.11 | 1.35±0.13 | 22.73 | 9.88 | 2.3 |
| IL-5 | 9.79±1.81 | 5.2±0.88 | 1.03±0.39 | 1.02±0.17 | 8.76 | 4.18 | 2.09 |
| IL-6 | 46.3±8.74 | 28.03±6.16 | 2.61±1.04 | 3.34±0.49 | 43.69 | 24.69 | 1.77 |
| IL-7 | 19.12±4.93 | 6.84±1.11 | 2.25±0.65 | 1.83±0.31 | 16.87 | 5.01 | 3.37 |
| IL-8 | 78.87±19.18 | 36.56±5.21 | 39.29±25.43 | 35.91±9.79 | 39.59 | 0.66 | **60.53** |
| IL-9 | 125.73±35.85 | 46.8±7.21 | 3.23±0.57 | 3.92±0.96 | 122.51 | 42.89 | 2.85 |
| IL-10 | 70.89±15.79 | 38.92±15.93 | 3.13±0.99 | 4.21±0.71 | 67.76 | 34.71 | 1.95 |
| IL-12(p70) | 49.88±8.18 | 20.09±4.38 | 3.9±0.69 | 3.49±0.66 | 45.99 | 16.59 | 2.77 |
| IL-13 | 44.72±7.27 | 23.9±9.02 | 1.18±0.18 | 1.58±0.19 | 43.54 | 22.33 | 1.95 |
| IL-15 | 63.33±20.31 | 37.32±7.89 | 4.06±0.61 | 7.39±1.88 | 59.29 | 29.92 | 1.98 |
| IL-17 | 42.1±9.71 | 43.74±17.43 | 1.61±0.23 | 3.26±1.34 | 40.39 | 40.48 | 0.99 |
| CCL11 | 109.64±15.72 | 56.37±7.84 | 12.65±4.22 | 19.71±3.5 | 96.99 | 36.66 | 2.65 |
| FGF basic | 22.17±2.94 | 15.11±2.75 | 1.32±0.35 | 1.27±0.58 | 20.86 | 13.85 | 1.5 |
| G-CSF | 34.56±2.64 | 30.39±3.99 | 7.97±1.44 | 8.23±0.82 | 26.59 | 22.16 | 1.2 |
| GM-CSF | 29.83±6.17 | 12.25±2.58 | 3.59±1.13 | 1.08±0.18 | 26.23 | 11.18 | 2.38 |
| IFN-γ | 110.36±23.59 | 85.04±13.47 | 23.36±4.02 | 46.37±8.24 | 86.99 | 38.68 | 2.25 |
| CXCL10 | 3996.39±528.36 | 2661.6±539.23 | 43.35±8.69 | 57.49±12.29 | 3953.04 | 2604.12 | 1.52 |
| CCL2 | 63.69±9.38 | 132.32±65.17 | 11.09±1.33 | 15.87±2.58 | 52.59 | 116.45 | 0.45 |
| CCL3 | 59.79±12.64 | 27.82±8.62 | 0.66±0.33 | 1.43±0.68 | 59.13 | 26.39 | 2.24 |
| PDGF-bb | 11874.59±9191.42 | 1799.9±346.92 | 112.99±21.93 | 191.23±48.41 | 11761.59 | 1608.67 | 7.31 |
| CCL4 | 1263.66±211.24 | 613.85±142.44 | 6.98±1.53 | 15.15±4.64 | 1256.68 | 598.71 | 2.09 |
| CCL5 | 3794.94±551.14 | 1835.77±493.28 | 51.94±10.14 | 74.37±15.43 | 3743.01 | 1761.4 | 2.13 |
| TNFα | 40.58±10.37 | 48.83±17.18 | 2.27±0.68 | 6.98±1.44 | 38.31 | 41.86 | 0.916 |
| VEGF | 225.07±38.03 | 92.72±17.27 | 11.85±2.07 | 20.04±5.18 | 213.22 | 72.68 | 2.94 |
| IL-1α | 0.6±0.1 | 0.66±0.14 | 1.44±0.16 | 1.09±0.19 | -0.83 | -0.44 | **1.91** |
| IL-2Ra | 142.59±20.24 | 119.45±21.94 | 32.51±4.58 | 23.48±4.17 | 110.08 | 95.98 | 1.15 |
| IL-3 | 203.89±38.82 | 196.88±26.69 | 77.52±8.39 | 50.78±6.97 | 126.38 | 146.11 | 0.87 |
| IL-12p40 | 290.01±47.8 | 286.42±36.36 | 107.69±18.73 | 59.18±12.05 | 182.31 | 227.25 | 0.81 |
| IL-16 | 168.8±16.42 | 292.6±88.32 | 114.63±10.26 | 133.94±19.43 | 54.191 | 158.66 | 0.35 |
| IL-18 | 28.31±4.51 | 25.91±3.48 | 9.45±1.94 | 7.43±2.32 | 18.89 | 18.48 | 1.02 |
| CCL27 | 63.06±8.29 | 81.33±12.63 | 138.66±12.79 | 105.29±15.42 | -75.59 | -23.97 | **3.16** |
| CXCL1 | 35.25±5.56 | 63.59±23.31 | 46.94±9.48 | 20.53±4.48 | -11.69 | 43.06 | -0.28 |
| HGF | 406.98±48.55 | 394.13±59.34 | 116.62±19.71 | 68.48±15.37 | 290.36 | 325.67 | 0.89 |
| IFN-α2 | 17.47±1.82 | 28.44±8.54 | 10.68±1.26 | 6.3±1.02 | 6.79 | 22.14 | 0.31 |
| LIF | 6.14±1.69 | 13.09±6.58 | 2.78±0.69 | 1.84±0.3 | 3.37 | 11.26 | 0.29 |
| CCL7 | 27.99±3.74 | 31.02±3.9 | 25.08±4.64 | 9.34±1.97 | 2.91 | 21.69 | 0.14 |
| M-CSF | 4.38±0.61 | 8.22±5.34 | 1.79±0.18 | 1.05±0.19 | 2.59 | 7.17 | 0.36 |
| MIF | 413.21±54.39 | 693.43±148.52 | 172.31±38.31 | 104.97±26.54 | 240.89 | 588.46 | 0.41 |
| CXCL9 | 1549.65±189.83 | 2211.09±596.85 | 145.27±28.71 | 93.12±18.96 | 1404.39 | 2117.97 | 0.66 |
| b-NGF | 4.81±1.09 | 14.79±10.45 | 0.83±0.09 | 0.59±0.07 | 3.99 | 14.19 | 0.28 |
| SCF | 63.79±8.18 | 86.37±12.78 | 35.74±3.48 | 30.87±2.96 | 28.06 | 55.49 | 0.51 |
| SCGF-b | 8108.39±851.25 | 9119.35±1842.39 | 1996.68±378.07 | 926.24±150.76 | 6111.71 | 8193.11 | 0.75 |
| CXCL12 | 59.48±7.61 | 74.42±9.88 | 65.41±5.93 | 30.89±6.29 | -5.93 | 43.52 | -0.14 |
| TNFβ | 0.79±0.09 | 4.61±3.07 | 1.39±0.43 | 0.9±0.22 | -0.59 | 3.7 | -0.17 |
| TRAIL | 39.94±5.61 | 49.61±8.04 | 18.82±3.67 | 12.83±3.03 | 21.12 | 36.79 | 0.58 |

- For arithmetic differences between mean cytokine levels in young and older subjects

Green color – positive values in green indicate that the mean was higher in patients compared with controls

Red color – negative values in red indicate that the mean was lower in patients compared with controls

** in this column we show the ratio of the 2 preceding columns ([younger patients minus controls] divided by [older patients minus controls])

Green color – positive values greater than 1 in green indicate that younger subjects responded better than older subjects (e.g. a value of 2 would indicate that the younger subjects adjusted by their control group, responded twice as intensively as older subjects adjusted for their control group

Red color

- positive values lower than 1 in red indicate that younger subjects responded less intensively than older subjects relative to their controls
- negative values in red indicate that whereas younger subjects had levels that were lower than their respective age-matched controls, older subjects had higher values relative to their age-matched controls

Purple colour- positive values in purple, in two cases, both younger and older subjects had lower levels than their respective age matched controls, but the younger groups had much lower levels relative to their age-matched controls than the older group.

Blue color – one example of a substantial differences in cytokine level between two groups is emphasized in blue. Here female subjects had only very marginally raised levels above their respective controls, but in males the difference was marked.

Supplemental Table 3. Analysis of serum cytokines in younger (≤40 years old) and older (>40 years old) NE male.

| **Cytokine** | **Patients (n=80)** | **Patients (n=37)** | **Controls (n=13)** | **Controls (n=8)** | **Arithmetic difference*** | | **X difference**** |
| --- | --- | --- | --- | --- | --- | --- | --- |
|  | **younger (40years and less)** | **older (>40 years)** | **younger (40 years and less)** | **older (>40 years)** | **Patients - controls** | | **younger/ older** |
|  |  |  |  |  | **younger** | **older** |  |
| IL-1β | 21.12±3.27 | 8.63±1.95 | 0.33±0.09 | 1.14±0.34 | 20.79 | 7.48 | 2.78 |
| IL-1ra | 165.49±49.78 | 135.52±41.29 | 17.39±6.18 | 51.3±118.56 | 148.09 | 84.21 | 1.76 |
| IL-2 | 39.09±16.43 | 16.46±3.04 | 1.14±0.14 | 1.38±0.35 | 37.96 | 15.08 | 2.52 |
| IL-4 | 24.08±3.94 | 11.44±2.95 | 0.94±0.11 | 1.48±0.19 | 23.14 | 9.94 | 2.33 |
| IL-5 | 10.58±1.95 | 4.78±0.95 | 0.57±0.15 | 1.2±0.19 | 10.01 | 3.58 | 2.79 |
| IL-6 | 47.84±9.27 | 25.79±6.24 | 1.65±0.31 | 3.59±0.85 | 46.19 | 22.19 | 2.08 |
| IL-7 | 20.61±5.326 | 7.47±1.29 | 1.21±0.37 | 1.85±0.52 | 19.41 | 5.61 | 3.45 |
| IL-8 | 84.41±20.75 | 37.19±6.23 | 11.78±5.38 | 30.92±6.65 | 72.64 | 6.26 | 11.58 |
| IL-9 | 134.66±38.83 | 46.58±8.83 | 4.04±1.21 | 4.69±1.75 | 130.62 | 41.89 | 3.11 |
| IL-10 | 75.65±17.07 | 44.64±22.22 | 2.26±0.61 | 5.72±1.24 | 73.39 | 38.92 | 1.88 |
| IL-12(p70) | 49.71±8.15 | 20.38±5.87 | 2.53±0.52 | 3.97±1.48 | 47.18 | 16.41 | 2.87 |
| IL-13 | 45.05±7.55 | 27.68±12.55 | 1.01±0.15 | 1.74±0.35 | 44.04 | 25.93 | 1.69 |
| IL-15 | 65.38±21.93 | 29.9±6.66 | 3.75±1.24 | 9.35±3.17 | 61.61 | 20.55 | 2.99 |
| IL-17 | 35.49±4.81 | 54.16±24.22 | 1.36±0.18 | 2.13±0.68 | 34.14 | 52.03 | 0.65 |
| CCL11 | 107.78±14.49 | 60.13±10.2 | 9.19±5.4 | 20.46±5.02 | 98.59 | 39.66 | 2.48 |
| FGF basic | 22.09±3.06 | 14.13±3.16 | 1.38±0.43 | 2.27±1.65 | 20.72 | 11.86 | 1.74 |
| G-CSF | 34.92±2.79 | 29.61±4.13 | 8.14±1.69 | 8.88±1.64 | 26.77 | 20.73 | 1.29 |
| GM-CSF | 32.32±6.64 | 10.74±2.59 | 5.01±2.51 | 1.1±80.37 | 27.30 | 9.55 | 2.85 |
| IFN-γ | 112.43±25.34 | 100.5317.99 | 21.24±4.28 | 61.01±16.19 | 91.19 | 39.51 | 2.3 |
| CXCL10 | 4330.38±559.39 | 2060.02±436.26 | 31.33±7.64 | 58.01±17.88 | 4299.05 | 2002.01 | 2.14 |
| CCL2 | 65.96±10.09 | 82.78±17.89 | 12.33±2.29 | 20.98±6.08 | 53.63 | 61.80 | 0.86 |
| CCL3 | 64.49±13.62 | 24.24±9.18 | 0.35±0.1 | 0.87±0.34 | 64.14 | 23.35 | 2.79 |
| PDGF-bb | 12745.81±9993.81 | 2057.75±461.65 | 78.61±12.04 | 312.85±120.38 | 12667.2 | 1744.9 | 7.26 |
| CCL4 | 1363.91±226.26 | 506.25±139.31 | 4.4±0.89 | 24.55±12.37 | 1359.5 | 481.69 | 2.82 |
| CCL5 | 4071.99±587.72 | 1820.03±561.58 | 37.68±7.59 | 114.51±39.76 | 4034.31 | 1705.52 | 2.36 |
| TNFα | 39.03±10.39 | 44.94±17.29 | 1.81±0.83 | 9.01±2.93 | 37.21 | 35.93 | 1.03 |
| VEGF | 225.47±38.72 | 100.69±22.51 | 8.99±3.49 | 21.26±8.99 | 216.47 | 79.44 | 2.72 |
| IL-1α | 0.64±0.1 | 0.74±0.17 | 1.38±0.28 | 1.11±0.33 | -0.75 | -0.36 | **2.07** |
| IL-2Ra | 140.37±21.91 | 102.75±23.43 | 38.41±9.72 | 31.29±10.39 | 101.94 | 71.46 | 1.42 |
| IL-3 | 206.01±42.1 | 206.42±35.91 | 70.22±8.01 | 50.49±11.27 | 135.79 | 155.93 | 0.87 |
| IL-12p40 | 297.2±51.7 | 283.47±47.18 | 98.48±21.78 | 55.91±18.76 | 198.72 | 227.56 | 0.87 |
| IL-16 | 171.5±17.54 | 334.06±123.13 | 117.62±12.56 | 177.94±49.15 | 53.93 | 156.13 | 0.34 |
| IL-18 | 24.73±2.77 | 23.71±3.61 | 7.01±1.96 | 8.75±5.92 | 17.72 | 14.96 | 1.18 |
| CCL27 | 57.94±8.1 | 78.37±13.77 | 127.29±16.94 | 112.09±32.56 | -69.33 | -33.71 | **2.05** |
| CXCL1 | 33.44±5.22 | 46.77±17.61 | 41.99±11.94 | 13.93±5.02 | -8.54 | 32.85 | -0.26 |
| HGF | 417.23±51.93 | 389.99±70.81 | 126.85±35.91 | 96.64±34.98 | 290.39 | 293.35 | 0.98 |
| IFN-α2 | 17.96±1.94 | 21.07±2.52 | 9.65±1.46 | 8.00±1.77 | 8.32 | 15.39 | 0.54 |
| LIF | 6.52±1.84 | 14.73±9.16 | 3.04±1.20 | 1.81±0.36 | 3.48 | 12.92 | 0.26 |
| CCL7 | 28.69±4.01 | 30.06±4.63 | 26.59±7.05 | 7.53±2.04 | 2.09 | 22.53 | 0.093 |
| M-CSF | 4.51±0.65 | 3.07±0.35 | 1.82±0.35 | 0.92±0.33 | 2.69 | 2.15 | 1.24 |
| MIF | 437.08±58.32 | 704.12±182.59 | 231.05±77.81 | 85.68±21.65 | 206.03 | 618.44 | 0.33 |
| CXCL9 | 1648.13±202.47 | 1885.03±389.93 | 134.06±50.67 | 115.67±45.65 | 1514.07 | 1769.36 | 0.85 |
| b-NGF | 3.9±0.89 | 18.24±14.64 | 0.83±0.09 | 0.66±0.15 | 3.07 | 17.58 | 0.17 |
| SCF | 61.44±8.12 | 73.62±10.83 | 33.62±4.31 | 33.49±5.067 | 27.83 | 40.13 | 0.69 |
| SCGF-b | 8545.47±908.81 | 11324.78±2505.85 | 1427.19±377.87 | 1008.24±282.6 | 7118.28 | 10316.55 | 0.68 |
| CXCL12 | 54.36±7.10 | 68.44±9.74 | 70.48±10.73 | 24.69±10.14 | -16.12 | 43.76 | -0.36 |
| TNFβ | 0.8±0.1 | 0.79±0.09 | 0.96±0.18 | 0.8±0.35 | -0.15 | -0.01 | **15.2** |
| TRAIL | 41.94±5.99 | 47.74±9.41 | 13.87±3.89 | 6.89±2.71 | 28.06 | 40.85 | 0.68 |

- For arithmetic differences between mean cytokine levels in young and older subjects

Green color – positive values in green indicate that the mean was higher in patients compared with controls

Red color – negative values in red indicate that the mean was lower in patients compared with controls

** in this column we show the ratio of the 2 preceding columns ([younger patients minus controls] divided by [older patients minus controls])

Green color – positive values greater than 1 in green indicate that younger subjects responded better than older subjects (e.g. a value of 2 would indicate that the younger subjects adjusted by their control group, responded twice as intensively as older subjects adjusted for their control group

Red color

- positive values lower than 1 in red indicate that younger subjects responded less intensively than older subjects relative to their controls
- negative values in red indicate that whereas younger subjects had levels that were lower than their respective age-matched controls, older subjects had higher values relative to their age-matched controls

Purple colour- positive values in purple, in three cases, both younger and older subjects had lower levels than their respective age matched controls, but the younger group had much lower levels relative to their age-matched controls than the older group.

Supplemental Table 4. Analysis of serum cytokines younger (≤40 years old) and older (>40 years old) NE female

| **Cytokine** | **Patients (n=7)** | **Patients (n=15)** | **Controls (n=21)** | **Controls (n=15)** | **Arithmetic difference*** | | **X difference**** |
| --- | --- | --- | --- | --- | --- | --- | --- |
|  | **younger (40years and less)** | **older (>40 years)** | **younger (40 years and less)** | **older (>40 years)** | **Patients - controls** | | **younger/ older** |
|  |  |  |  |  | **younger** | **older** |  |
| IL-1β | 9.75±5.62 | 8.1±3.74 | 0.67±0.26 | 1.23±0.35 | 9.08 | 6.87 | 1.32 |
| IL-1ra | 49.5±12.19 | 72.06±16.83 | 20.27±5.96 | 48.65±13.95 | 29.23 | 23.4 | 1.24 |
| IL-2 | 22.5±10.92 | 10.42±3.6 | 2.5±0.77 | 1.55±0.24 | 19.99 | 8.87 | 2.25 |
| IL-4 | 19.03±11.08 | 10.69±4.24 | 0.95±0.16 | 1.27±0.16 | 18.08 | 9.42 | 1.91 |
| IL-5 | 0.71±0.24 | 6.23±2.04 | 1.31±0.61 | 0.92±0.22 | -0.6 | 5.3 | -0.11 |
| IL-6 | 29.03±25.03 | 33.55±15.09 | 3.2±1.67 | 3.2±0.62 | 25.83 | 30.35 | 0.85 |
| IL-7 | 2.02±0.48 | 5.28±2.15 | 2.89±0.99 | 1.81±0.39 | -0.87 | 3.46 | -0.25 |
| IL-8 | 15.56±7.11 | 35.01±9.82 | 56.3±40.96 | 38.56±14.75 | -40.74 | -3.54 | **11.48** |
| IL-9 | 23.71±11.11 | 47.33±12.7 | 2.71±0.51 | 3.5±1.16 | 20.98 | 43.83 | 0.47 |
| IL-10 | 16.42±8.45 | 24.78±7.09 | 3.66±1.55 | 3.39±0.8 | 12.751 | 21.39 | 0.59 |
| IL-12(p70) | 51.76±43.82 | 19.39±4.9 | 4.73±1.02 | 3.24±0.67 | 47.03 | 16.15 | 2.91 |
| IL-13 | 40.93±28.85 | 14.6±4.48 | 1.28±0.27 | 1.49±0.22 | 39.65 | 13.1 | 3.02 |
| IL-15 | 40.01±32.11 | 55.51±21.73 | 4.23±0.63 | 6.35±2.35 | 35.77 | 49.22 | 0.72 |
| IL-17 | 116.33±110.86 | 18.01±6.9 | 1.76±0.35 | 3.86±2.02 | 114.57 | 14.15 | 8.09 |
| CCL11 | 130.78±110.84 | 47.09±10.33 | 14.78±5.99 | 19.3±4.77 | 115.99 | 27.79 | 4.17 |
| FGF basic | 22.94±11.39 | 17.53±5.6 | 1.27±0.513 | 0.73±0.13 | 21.67 | 16.8 | 1.28 |
| G-CSF | 30.46±8.09 | 32.31±9.62 | 7.86±2.12 | 7.88±0.94 | 22.6 | 24.43 | 0.92 |
| GM-CSF | 1.42±0.33 | 15.96±6.28 | 2.72±0.99 | 1.01±0.19 | -1.3 | 14.95 | -0.08 |
| IFNγ | 86.59±47.65 | 46.82±9.45 | 24.67±6.00 | 38.55±8.99 | 61.91 | 8.27 | 7.48 |
| CXCL10 | 179.26±59.1 | 4145.5±1497.91 | 50.78±13.16 | 57.20±16.69 | 128.47 | 4088.29 | 0.03 |
| CCL2 | 37.58±15.23 | 254.49±223.81 | 10.31±1.65 | 13.14±2.15 | 27.26 | 241.35 | 0.11 |
| CCL3 | 6.01±4.78 | 36.69±19.87 | 0.85±0.53 | 1.729±1.03 | 5.15 | 34.96 | 0.14 |
| PDGF-bb | 1917.81±1716.40 | 1163.85±357.01 | 134.28±34.23 | 126.37±30.28 | 1783.52 | 1037.47 | 1.71 |
| CCL4 | 117.87±87.91 | 879.26±355.06 | 8.57±2.37 | 10.12±2.43 | 109.31 | 869.14 | 0.12 |
| CCL5 | 628.62±563.86 | 1874.57±1035.53 | 60.76±15.59 | 52.95±7.57 | 567.87 | 1821.62 | 0.31 |
| TNFα | 58.21±53.12 | 58.41±42.64 | 2.55±0.96 | 5.88±1.56 | 55.68 | 52.52 | 1.06 |
| VEGF | 220.47±178.71 | 73.02±22.57 | 13.61±2.55 | 19.38±6.49 | 206.87 | 53.64 | 3.85 |
| IL-1α | 0.3±0.08 | 0.42±0.14 | 1.47±0.19 | 1.07±0.23 | -1.16 | -0.66 | **1.75** |
| IL-2Ra | 167.96±23.87 | 160.63±49.26 | 28.85±4.36 | 19.31±3.09 | 139.1 | 141.33 | 0.98 |
| IL-3 | 179.58±38.77 | 173.32±27.56 | 82.03±12.71 | 50.93±9.13 | 97.56 | 122.39 | 0.79 |
| IL-12p40 | 207.76±58.11 | 293.69±50.65 | 113.39±27.51 | 60.92±15.97 | 94.37 | 232.78 | 0.41 |
| IL-16 | 137.55±38.97 | 190.29±35.74 | 112.77±14.89 | 110.48±12.34 | 24.78 | 79.82 | 0.31 |
| IL-18 | 69.15±46.51 | 31.34±8.16 | 10.95±2.87 | 6.73±1.86 | 58.21 | 24.61 | 2.36 |
| CCL27 | 121.38±41.73 | 88.59±28.39 | 145.69±18.01 | 101.66±16.95 | -24.31 | -13.08 | **1.85** |
| CXCL1 | 55.86±36.29 | 105.03±68.69 | 50.01±13.61 | 24.05±6.18 | 5.87 | 80.99 | 0.07 |
| HGF | 289.67±107.26 | 404.38±112.44 | 110.28±23.51 | 53.45±14.01 | 179.39 | 350.92 | 0.51 |
| IFN-α2 | 11.85±3.75 | 46.6±29.09 | 11.31±1.84 | 6.65±±1.29 | 0.55 | 39.95 | 0.01 |
| LIF | 1.71±0.47 | 9.04±3.601 | 2.6±0.84 | 1.850.44 | -0.89 | 7.19 | -0.13 |
| CCL7 | 19.84±7.55 | 33.38±7.33 | 24.13±6.22 | 10.3±2.83 | -4.29 | 23.08 | -0.19 |
| M-CSF | 2.7±0.59 | 20.89±18.49 | 1.77±0.18 | 1.13±0.25 | 0.94 | 19.77 | 0.05 |
| MIF | 140.26±42.43 | 667.03±258.86 | 135.95±38.71 | 115.26±39.38 | 4.32 | 551.77 | 0.01 |
| CXCL9 | 424.11±143.05 | 3015.35±1862.59 | 152.19±35.24 | 81.16±16.76 | 271.91 | 2934.24 | 0.09 |
| b-NGF | 15.16±8.52 | 6.26±3.06 | 0.82±0.13 | 0.57±0.07 | 14.34 | 5.69 | 2.52 |
| SCF | 90.61±43.01 | 117.79±34.91 | 37.05±5.01 | 29.48±3.73 | 53.57 | 88.31 | 0.6 |
| SCGF-b | 3113.06±526.49 | 3679.28±259.11 | 2349.22±559.05 | 882.5±181.87 | 763.84 | 2796.77 | 0.27 |
| CXCL12 | 117.93±45.91 | 89.14±24.68 | 62.26±7.05 | 34.21±8.12 | 55.67 | 54.92 | 1.01 |
| TNFβ | 0.63±0.12 | 14.02±10.49 | 1.65±0.68 | 0.97±0.28 | -1.03 | 13.05 | -0.07 |
| TRAIL | 17.01±9.48 | 54.21±15.84 | 21.87±5.39 | 15.99±4.25 | -4.86 | 38.22 | -0.12 |

- For arithmetic differences between mean cytokine levels in young and older subjects

Green color – positive values in green indicate that the mean was higher in patients compared with controls

Red color – negative values in red indicate that the mean was lower in patients compared with controls

** in this column we show the ratio of the 2 preceding columns ([younger patients minus controls] divided by [older patients minus controls])

Green color – positive values greater than 1 in green indicate that younger subjects responded better than older subjects (e.g. a value of 2 would indicate that the younger subjects adjusted by their control group, responded twice as intensively as older subjects adjusted for their control group

Red color

- positive values lower than 1 in red indicate that younger subjects responded less intensively than older subjects relative to their controls
- negative values in red indicate that whereas younger subjects had levels that were lower than their respective age-matched controls, older subjects had higher values relative to their age-matched controls

Purple colour- positive values in purple, in three cases, both younger and older subjects had lower levels than their respective age matched controls, but the younger group had much lower levels relative to their age-matched controls than the older group.
